# A deep learning-powered diagnostic model for acute pancreatitis

**DOI:** 10.1186/s12880-024-01339-9

**Published:** 2024-06-20

**Authors:** Chi Zhang, Jin Peng, Lu Wang, Yu Wang, Wei Chen, Ming-wei Sun, Hua Jiang

**Affiliations:** 1 Department of Intensive Care Medicine, The Third People’s Hospital of Chengdu, Affiliated Hospital of Southwest Jiaotong University, Chengdu, China; 2 Institute for Emergency and Disaster Medicine, School of Medicine, Sichuan Provincial People’s Hospital, University of Electronic Science and Technology of China, Chengdu, China; 3 Department of Emergency Medicine, School of Medicine, Sichuan Provincial People’s Hospital, University of Electronic Science and Technology of China, Chengdu, China; 4 Sichuan Provincial Clinical Research Center for Emergency and Critical Care, School of Medicine, University of Electronic Science and Technology of China, Chengdu, China; 5 Department of Histology and Neuroscience, Sichuan University, Chengdu, China; 6 Department of Clinical Nutrition, Peking Union Medical College Hospital, Beijing, China

**Keywords:** Acute pancreatitis, Diagnosis, Abdominal CT, Deep learning, Semantic segmentation

## Abstract

**Background:**

Acute pancreatitis is one of the most common diseases requiring emergency surgery. Rapid and accurate recognition of acute pancreatitis can help improve clinical outcomes. This study aimed to develop a deep learning-powered diagnostic model for acute pancreatitis.

**Materials and methods:**

In this investigation, we enrolled a cohort of 190 patients with acute pancreatitis who were admitted to Sichuan Provincial People’s Hospital between January 2020 and December 2021. Abdominal computed tomography (CT) scans were obtained from both patients with acute pancreatitis and healthy individuals. Our model was constructed using two modules: (1) the acute pancreatitis classifier module; (2) the pancreatitis lesion segmentation module. Each model’s performance was assessed based on precision, recall rate, F1-score, Area Under the Curve (AUC), loss rate, frequency-weighted accuracy (fwavacc), and Mean Intersection over Union (MIOU).

**Results:**

Upon admission, significant variations were observed between patients with mild and severe acute pancreatitis in inflammatory indexes, liver, and kidney function indicators, as well as coagulation parameters. The acute pancreatitis classifier module exhibited commendable diagnostic efficacy, showing an impressive AUC of 0.993 (95%CI: 0.978–0.999) in the test set (comprising healthy examination patients vs. those with acute pancreatitis, *P* < 0.001) and an AUC of 0.850 (95%CI: 0.790–0.898) in the external validation set (healthy examination patients vs. patients with acute pancreatitis, *P* < 0.001). Furthermore, the acute pancreatitis lesion segmentation module demonstrated exceptional performance in the validation set. For pancreas segmentation, peripancreatic inflammatory exudation, peripancreatic effusion, and peripancreatic abscess necrosis, the MIOU values were 86.02 (84.52, 87.20), 61.81 (56.25, 64.83), 57.73 (49.90, 68.23), and 66.36 (55.08, 72.12), respectively. These findings underscore the robustness and reliability of the developed models in accurately characterizing and assessing acute pancreatitis.

**Conclusion:**

The diagnostic model for acute pancreatitis, driven by deep learning, exhibits excellent efficacy in accurately evaluating the severity of the condition.

**Trial Registration:**

This is a retrospective study.

## Background

Acute pancreatitis is one the most common diseases in emergency departments and is characterized by local and systemic inflammation with different clinical courses [1, 2]. The symptoms of acute pancreatitis are non-specific and may include abdominal pain, nausea, vomiting and fever. These symptoms can be difficult to distinguish from those of other gastrointestinal diseases, such as cholecystitis, acute gastroenteritis and acute appendicitis. Furthermore, acute pancreatitis may present with atypical symptoms, such as back pain, which can result in incorrect or delayed diagnosis. Although elevated serum amylase and lipase levels are characteristic of acute pancreatitis, these enzymes can also be elevated under other conditions, which can lead to false-positive results. In 2017, there were about 1.6 million new cases of acute pancreatitis worldwide, of which about 100,000 resulted in death [3]. Acute pancreatitis is mostly self-limited. However, around 20% of the patients develop acute severe pancreatitis and the death rate is about 30% [4]. Although several models have been developed to predict pancreatitis-related outcomes, their accuracy is unsatisfactory [5, 6]. At the present, there are many clinical scoring systems for the early classification of acute pancreatitis severity, among which Acute Physiological and Chronic Health Score (APACHE) II and Acute Pancreatitis Severity Bed Side Index (BISAP) are widely used in clinical practice [7]. The BISAP score can be evaluated on the first day of admission, but the accuracy and sensitivity of its prediction are not high [8]. In imaging, the assessment of acute pancreatitis relies on the Balthazar CT [9] rating and the Modified Computed Tomography Severity Index Score (MCTSI) [10]. However, in early stages of acute pancreatitis, morphological changes of the pancreas may not be apparent on CT or MRI images in some patients, especially pancreatic necrosis, which may lead to underestimation of the severity of the disease [11, 12]. The severity of symptoms and manifestations of acute pancreatitis varies from person to person, and it can result in complications, including the formation of pseudocysts and organ failure. The early recognition of these complications is of the utmost importance for the appropriate management of the condition and the improvement of patient outcome.

Recently, artificial intelligence (AI) is poised to revolutionize the future development of medicine [13]. Through AI models, an accurate prediction of results can be achieved by learning complex relationships among the data presented [14]. With the advancement of computing technologies and the development of medical databases, machine learning has become an active area of medical research. Machine learning in medicine can generate more accurate diagnostic algorithms and individualized patient treatment plans [15, 16]. In recent years, AI has also been widely applied to acute pancreatitis diagnosis and treatment, especially in severity evaluation [17–19], complications [20–22], mortality [23, 24], recurrence [25, 26], and surgery time prediction [27, 28] with various degrees of breakthroughs.

Traditional machine learning (ML) constructs models for diagnosis and predictions based on clinical and laboratory data of patients with acute pancreatitis. Deep learning (DL), as a primary research direction in the field of ML, has its unique advantages. DL can learn patterns and features within data, and the information obtained in the process of learning can be made highly interpretable for numerical, image and other data. The Convolutional Neural Networks (CNN) is a class of feedforward neural networks that uses convolutional computation with a deep neural network structure and is one of the representative algorithms of DL [29]. Among the different variants of CNN-based networks, U-Net has become one of the main choices, a network model proposed by Ronneberger et al. [30] in 2015, which consists of a symmetric encoder-decoder network with hopping connections for enhanced detail retention. The U-Net network was used for semantic segmentation of medical imaging data when it was proposed and was extended to semantic segmentation of 3D video data [31] and generation of super-resolution images [32] in subsequent applied research. Numerous studies [33–40] have proposed novel model architectures based on the U-net model architecture, which have demonstrated significant advancements in image segmentation performance and model parameter requirements. EasyDL is a DL platform developed by Baidu company that allows researchers to create and train models easily [41]. EasyDL’s underlying layers combine AutoDL and AutoML technologies to automatically obtain optimal networks, which avoids tedious network selection and hyperparameter tuning for non-specialists in building DL models. Therefore, this study merges two autonomous modules to develop an ensemble learning model, which not only improves the performance of the model, but also enhances its interpretability. Previous research in the diagnosis and prediction of acute pancreatitis has only been conducted at the level of traditional ML [17–28]. The advantages of DL have not been effectively utilized, and this study proposes to construct a unique diagnostic prediction model for acute pancreatitis through DL methods.

To that end, we broke into new territories in the following areas: (1) This is the first instance in which DL has been employed to construct a diagnostic model for acute pancreatitis. (2) We innovatively combined the acute pancreatitis classifier and lesion segmentation modules to construct a diagnostic model, which can not only quickly identify pancreatitis, but also identify pancreatitis-associated foci and directly assess the severity of the disease.

This paper is structured as follows: Sect. 2 outlines the methodology employed in this study. This includes an overview of the inclusion and exclusion criteria, the data collection and processing process, and the model construction. The model was made up of two modules: (1) the acute pancreatitis classifier module; (2) the pancreatitis lesion segmentation module. The statistical methods employed, and the evaluation methods used to assess the final model. Section 3 presents the results section of this study, including the statistical results of the patients’ baseline data and the results of the modelling. Section 4 is devoted to the strengths of the model and some of the current limitations. It concludes with a discussion of future directions for improving the study.

## Materials and methods

### Patients

The protocol of this retrospective study was approved by the Medical Ethics Committee of Sichuan Provincial People’s Hospital, with consent information waived (approval number: Ethical Review (Research) No. 99 of 2022). The investigation involved patients admitted to the emergency medicine center of Sichuan Provincial People’s Hospital for acute pancreatitis from January 2020 to December 2021. Additionally, healthy control individuals from the Physical Examination Center of Sichuan Provincial People’s Hospital during the same period were included in the study. Inclusion and exclusion criteria are shown in Table 1.

**Table 1:** Inclusion and exclusion criteria

Inclusion criteria	Exclusion criteria
1. Patients age ≥ 18 years	1. Patients age < 18 years
2. Admitted to Sichuan Emergency Center for treatment of acute pancreatitis	2. No abdominal CT examination during hospitalization
3. At least one abdominal CT examination during hospitalization	3. Previous history of gastrointestinal tumors
4. The resolution of CT images is 512*512 pixels	4. Previous history of pancreatic surgery
5. Abdominal CT for health screening patients	5. Poor quality of CT image imaging

### Data collection

#### Clinical and laboratory data collection

Upon admission to the emergency department, comprehensive clinical and laboratory data were meticulously gathered. This encompassed demographic information such as gender and age, along with pertinent medical histories including hypertension, diabetes, smoking habits, and alcohol consumption. The laboratory dataset included a range of parameters: blood routine, inflammatory marker, liver and kidney function indicators, serum amylase, lipase, blood lipids, and coagulation function.

#### Imaging data collection

For the purposes of DL in this study, abdominal CT images served as the primary dataset. A meticulous process was followed, where two independent radiologists screened the abdominal CT images of both patients and healthy control individuals. Subsequently, images depicting noticeable pancreatic swelling indicative of acute pancreatitis or those exhibiting a normal pancreas in physical examinations were selected. The final step involved a thorough review of the screened images by a senior radiologist, ensuring the precision and reliability of the dataset for subsequent analyses.

### Development of classifier module for acute pancreatitis

In our research, we employed Baidu’s EasyDL platform (https://ai.baidu.com/easydl/) as the foundation for constructing a classifier module dedicated to acute pancreatitis. Adhering to EasyDL’s operational protocol, we uploaded CT images representing both acute pancreatitis and healthy pancreas, and these images were subsequently trained using EasyDL’s optimal network. We observed and recorded the performance metrics of the module during training. To comprehensively assess the module’s robustness, an untrained dataset was used for external validation, providing valuable insights into its generalization capabilities beyond the training dataset.

### Development of lesion segmentation module for acute pancreatitis

In this study, the delineation of pancreatic conditions, including normal pancreas, swollen pancreas, peripancreatic inflammatory exudate, peripancreatic effusion, and peripancreatic abscess necrosis, was anchored on the Balthazar CT rating. To execute this segmentation task, we used the open-source software Genie Annotation Assistant. Two radiologists performed pixel-level segmentation of the lesions, and subsequently, a senior radiologist reviewed the segmented content.

**Fig. 1:**
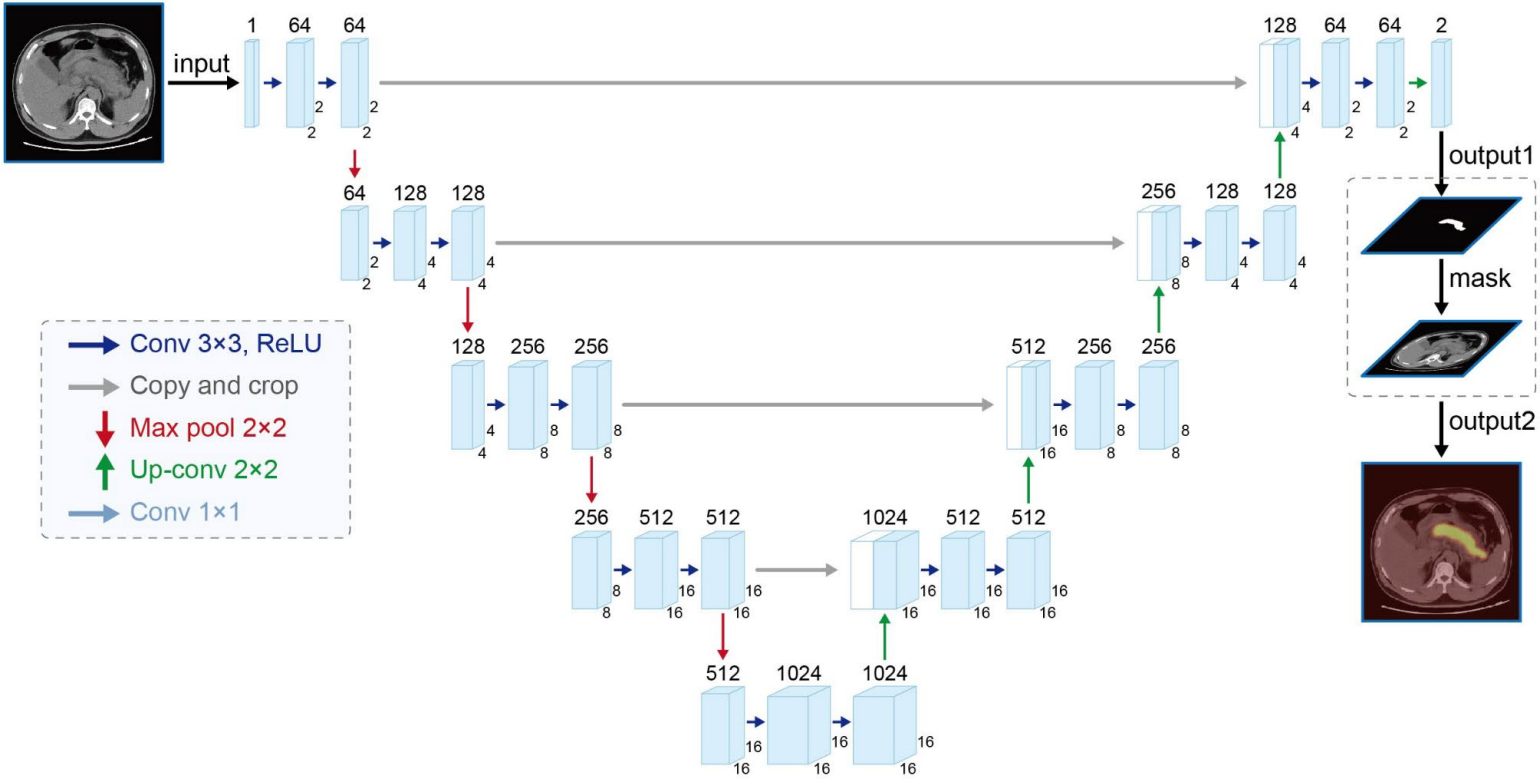
Structure of U-Net network

The U-Net network [30], illustrated in Fig. 1, was chosen as the foundational architecture for the segmentation module. This module exhibits a straightforward structure, with the left section devoted to feature extraction and the right part to up-sampling. Termed the Encoder-Decoder structure in the realm of research, the U-Net network maximizes the effectiveness of segmentation data utilization by employing a data enhancement method, particularly advantageous when dealing with a limited number of segmented images.

**Fig. 2:**
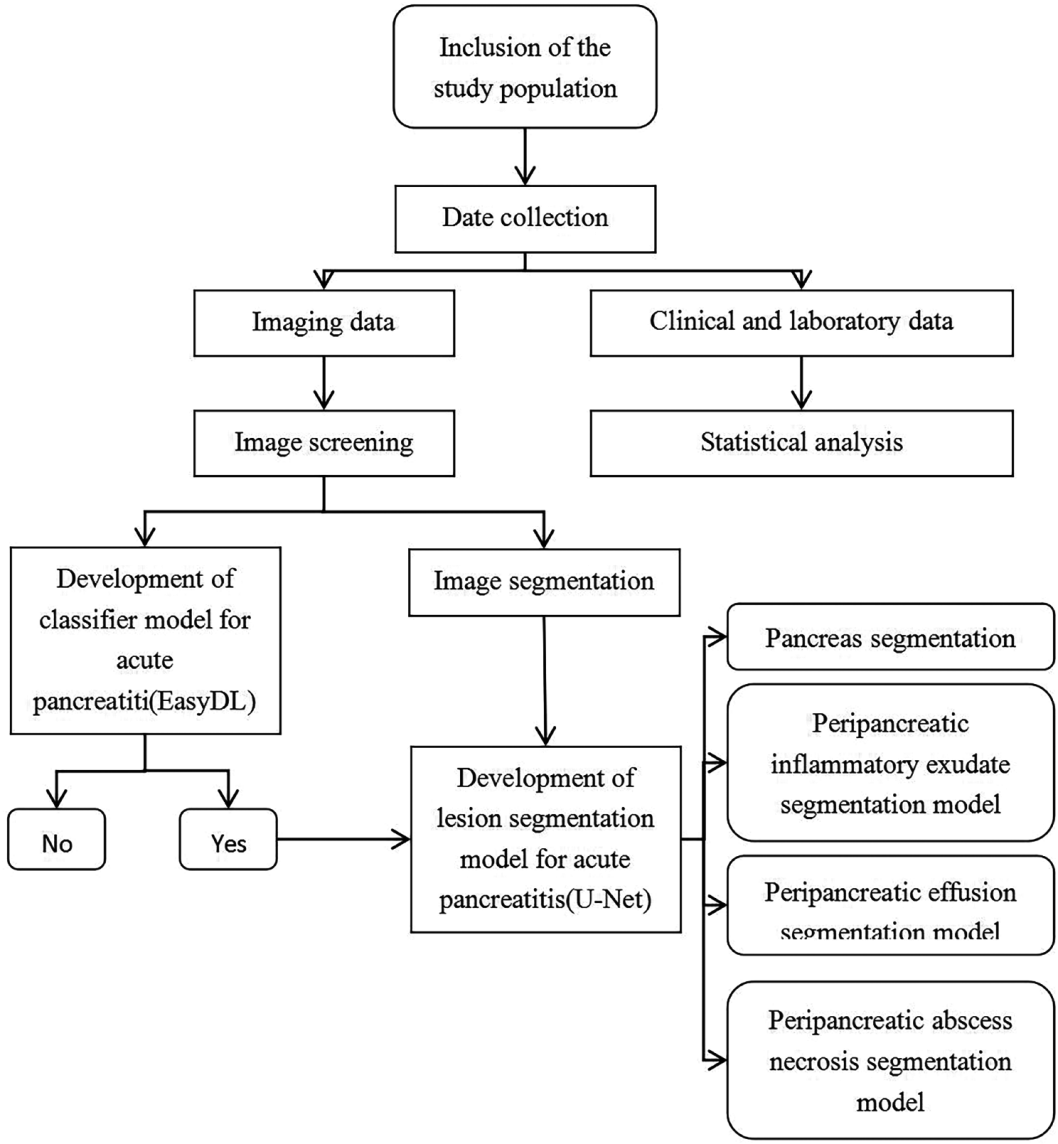
The workflow of the methodology

For optimal module training, the dataset was intelligently partitioned into a training set and a validation set through a computer-generated random division, maintaining an 8:2 ratio. This strategic division ensures robust training and reliable validation, contributing to the overall efficacy of the segmentation module. The workflow of the methodology is shown in Fig. 2.

### Statistical methods

For the analysis of small sample continuous data, the normality of the data distribution was assessed using the Shapiro-Wilk test. In instances where the data exhibited normal distribution, they were presented as mean ± standard deviation (± S), and inter-group comparisons were conducted using the independent sample t-test. Alternatively, for skewed data, representation was made using the median and quartile [M (P25, P75)], and inter-group comparisons were performed using the Mann-Whitney U test.

Dichotomous data underwent inter-group comparisons through Chi-square tests. The statistical analysis was executed using IBM SPSS Statistics 26.0 software (IBM, America).

### Model evaluation

In the acute pancreatitis classifier module, the assessment of module efficacy relied on the area under the Receiver Operating Characteristic (ROC) curve. The acute pancreatitis lesion segmentation module’s performance was gauged by analyzing the accuracy, loss rate, frequency-weighted accuracy (fwavacc), and Mean Intersection over Union (MIOU) across both the training and validation sets.

## Result

### Demographic characteristics

A total of 190 patients with acute pancreatitis were included in this study, of which 121 (63.68%) were males and 69 (36.32%) were females. According to the severity of the disease, 100 cases (52.63%) were classified as mild acute pancreatitis and 90 cases (47.37%) as severe acute pancreatitis (moderate and severe acute pancreatitis are defined as severe acute pancreatitis in this study). The clinical and laboratory data of patients with pancreatitis were collected in accordance with the methodology detailed in Table 2. The differences between the two groups in smoking, leukocytes, neutrophils, hematocrit (HCT), platelets (PLT), C-reactive protein (CRP), procalcitonin (PCT), urea, creatinine (Cr), glucose (Glu), calcium (Ca), albumin (ALB), aspartate transaminase (AST), lactate dehydrogenase (LDH), total bilirubin (TB), amylase (AMY), lipase (LPS), total cholesterol (TC), LDL, PT, APTT, DD, and FDP were statistically significant (*P* < 0.05).

**Table 2:** Baseline information on admission of patients with acute pancreatitis

Projects	Acute mild pancreatitis (*n* = 100)	Acute severe pancreatitis (*n* = 90)	*P* Value
Age	46.68 ± 13.43	50.29 ± 16.85	0.107
Gender (M/F)	63/37	58/32	0.836
High blood pressure	18	21	0.363
Diabetes	21	14	0.334
Smoking	47	27	**0.016**
Drinking	41	28	0.157
WBC (10^9^/L)	11.19 (8.35, 14.09)	13.04 (10.86, 17.36)	**0.001**
N (10^9^/L)	8.90 (6.41, 12.24)	11.79 (9.17, 15.41)	**0.000**
M (10^9^/L)	0.50 (0.30, 0.72)	0.56 (0.39, 0.77)	0.104
HB (g/L)	145.74 ± 21.74	150.82 ± 28.66	0.174
HCT (%)	42.15 (39.50, 46.58)	45.30 (38.93, 50.50)	**0.048**
PLT (10^9^/L)	187.00 (132.00, 239.75)	157.00 (124.75, 210.50)	**0.042**
CRP (mg/L)	21.72 (5.20, 95.14)	92.40 (10.45, 211.29)	**0.000**
PCT (ng/mL)	0.13 (0.05, 0.35)	0.81 (0.22, 3.04)	**0.000**
Urea (mmol/L)	4.14 (3.05, 5.29)	6.05 (3.96, 8.59)	**0.000**
Cr (umol/L)	56.90 (46.33, 64.98)	61.65 (52.28, 103.50)	**0.001**
GLU (mmol/L)	8.10 (5.98, 11.34)	9.02 (7.06, 13.39)	**0.022**
K^+^ (mmol/L)	3.93 (3.68, 4.14)	3.92 (3.66, 4.31)	0.521
Na^+^ (mmol/L)	137.80 (135.33, 139.76)	137.70 (134.70, 140.20)	0.734
Ca^2+^ (mmol/L)	2.22 (2.08, 2.36)	2.10 (1.92, 2.28)	**0.000**
ALB (g/L)	40.14 ± 5.97	36.40 ± 6.13	**0.000**
AST (U/L)	38.00 (27.25, 61.25)	62.50 (40.50, 165.00)	**0.000**
ALT (U/L)	36.50 (21.25, 66.75)	39.00 (22.75, 132.25)	0.166
LDH (U/L)	225.50 (194.00, 280.75)	420.00 (256.50, 551.00)	**0.000**
ALP (U/L)	89.00 (74.25, 120.50)	97.00 (73.50, 134.00)	0.232
TBIL (umol/L)	18.15 (13.93, 29.75)	26.20 (18.40, 47.83)	**0.000**
AMY (U/L)	197.50 (99.25, 694.00)	728.50 (229.25, 1455.50)	**0.000**
LPS (U/L)	318.25 (113.65, 1529.88)	1016.20 (287.58, 2667.88)	**0.001**
TC (mmol/L)	4.64 (3.72, 6.60)	4.12 (3.06, 5.40)	**0.007**
TG (mmol/L)	2.04 (1.06, 8.13)	1.79 (1.00, 4.05)	0.294
LDL (mmol/L)	2.01 (1.64, 2.79)	1.90 (1.33, 2.44)	**0.029**
HDL (mmol/L)	1.04 (0.82, 1.32)	1.07 (0.65, 1.33)	0.922
ApoA1/ApoB	1.30 (1.10, 1.80)	1.30 (1.00, 1.80)	0.398
PT (s)	11.65 (11.00, 12.50)	12.30 (11.55, 13.65)	**0.000**
APTT (s)	26.95 (25.83, 28.28)	27.90 (25.45, 30.85)	**0.018**
FIB (g/L)	3.72 (2.99, 5.28)	3.96 (3.00, 6.57)	0.199
D- Dimer (mg/L)	1.15 (0.43, 2.03)	3.25 (1.33, 6.41)	**0.000**
FDP (mg/L)	3.30 (2.50, 5.48)	7.70 (3.45, 15.60)	**0.000**

### Imaging data

**Fig. 3:**
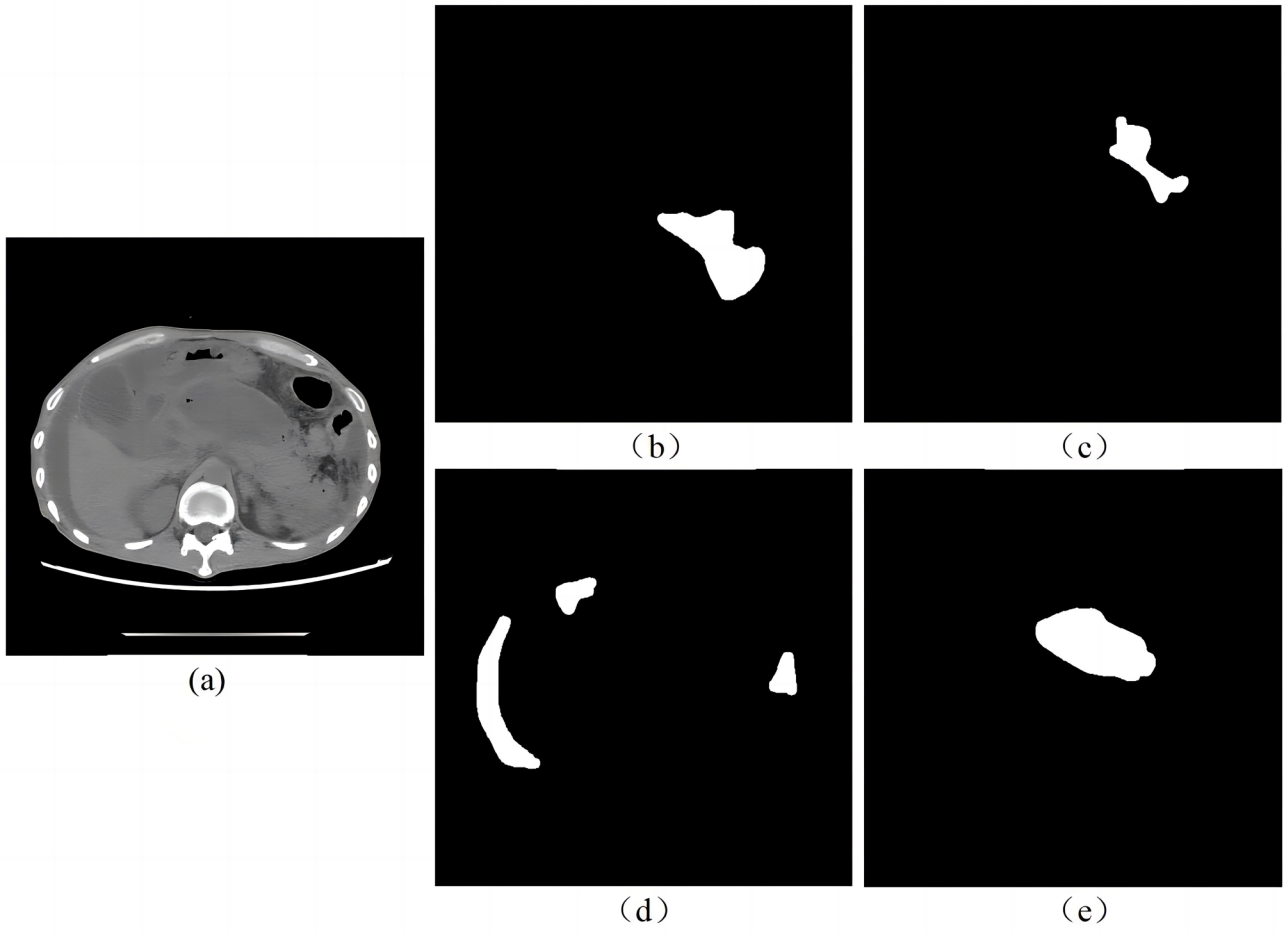
Lesion segmentation diagram. (**a**) original CT image of acute pancreatitis; (**b**) manual segmentation of swollen pancreas; (**c**) manual segmentation of peripancreatic inflammatory exudate; (**d**) manual segmentation of peripancreatic effusion; (**e**) manual segmentation of peripancreatic necrosis

Image study was performed by two radiologists outlining the target area and one senior radiologist reviewing the result. A total of 945 segmented images of swollen pancreas, 592 segmented images of normal pancreas, 475 segmented images of peripancreatic inflammatory exudate, 153 segmented images of peripancreatic effusion, and 42 segmented images of peripancreatic abscess necrosis were obtained. Figure 3 shows the lesion segmentation diagram.

### Classifier module for acute pancreatitis

**Fig. 4:**
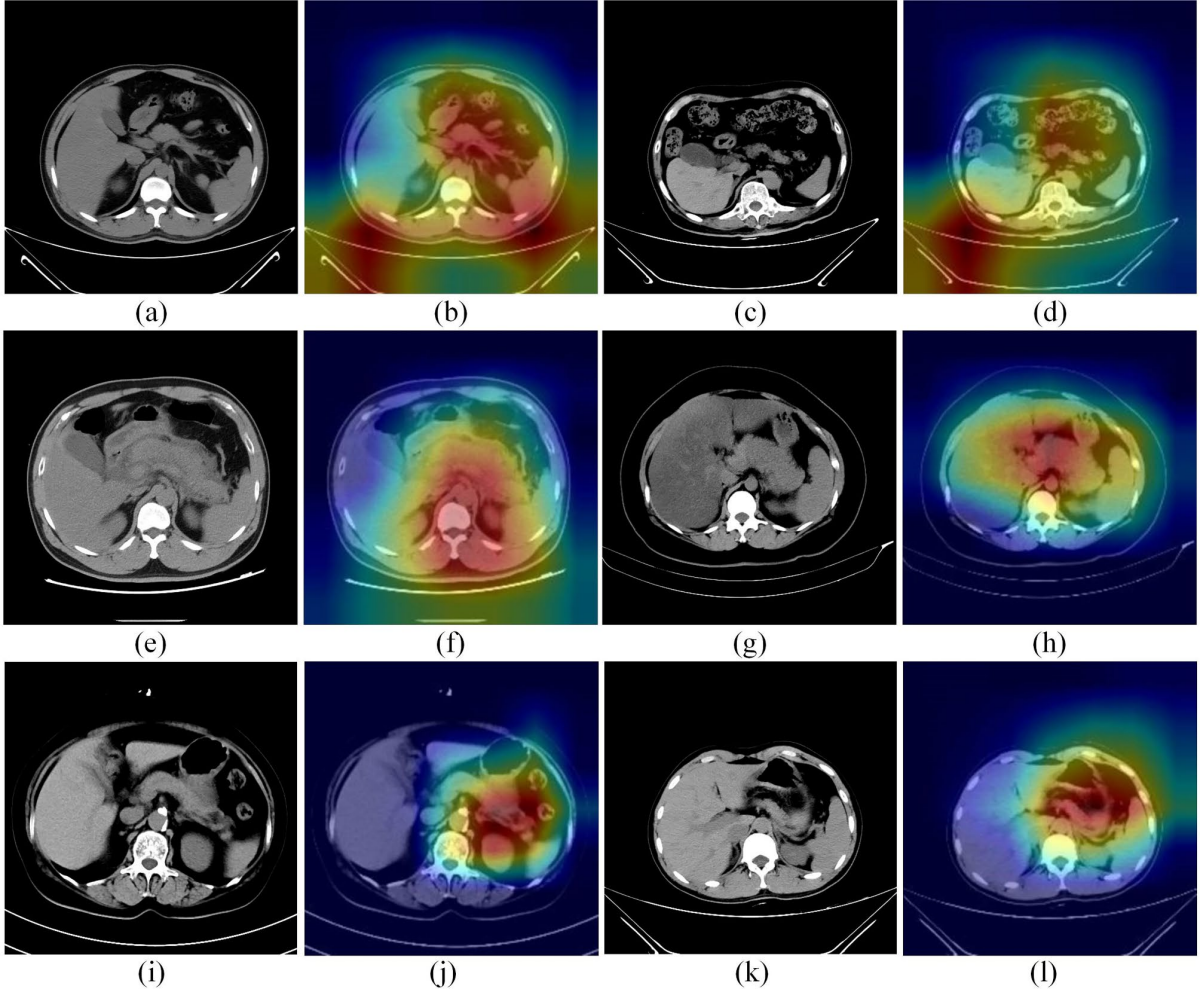
The heat map of the classifier module for acute pancreatitis

**Fig. 5:**
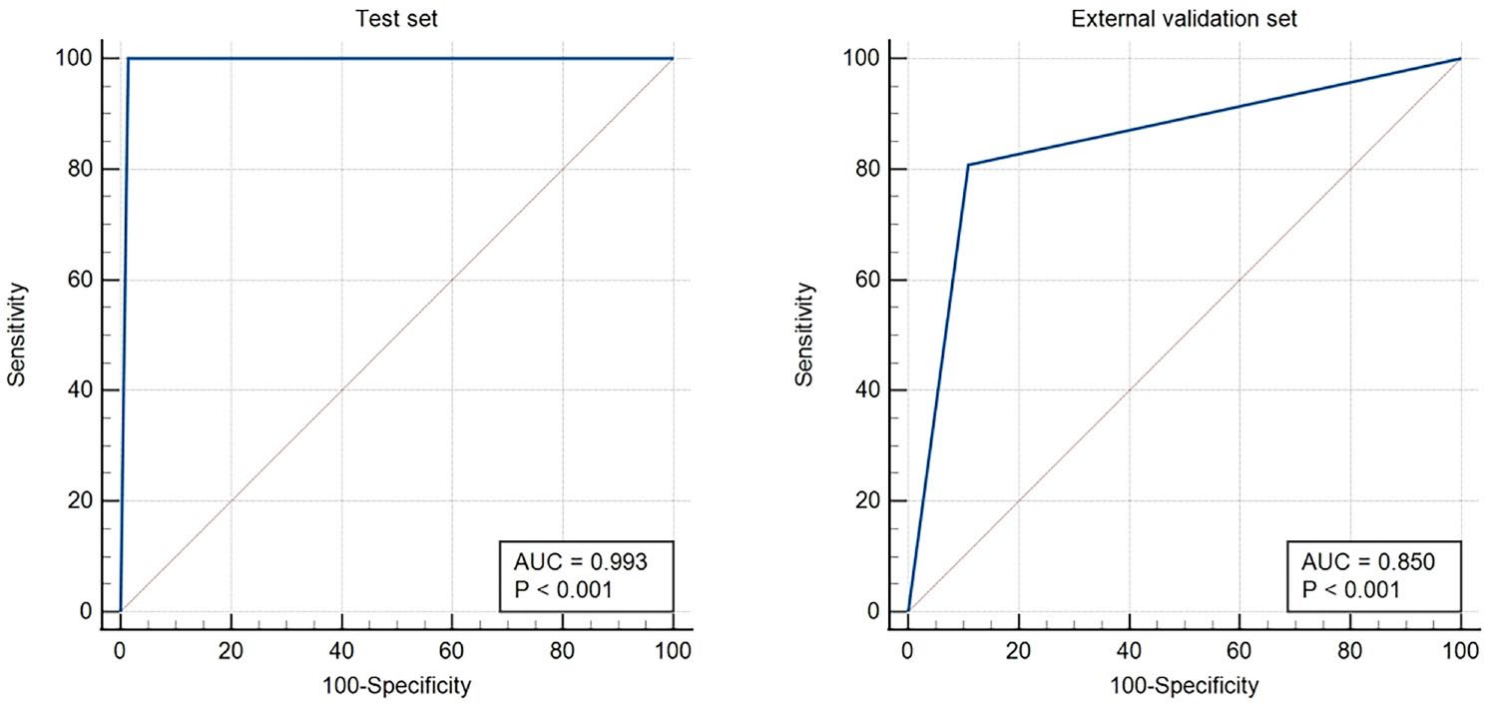
The ROC curves of the classifier module in the test set and external validation set

The classifier module obtained by EasyDL was highly effective, with 99.1% precision rate, 100% recall rate and 100% f1-score for predicting acute pancreatitis. Among 352 random samples, 350 were correctly predicted by the module and 2 samples were incorrectly predicted (Table 3). The heat map is shown in Fig. 4. To verify the performance of the module, 186 untrained images were selected and inputted to EasyDL for validation. The ROC curves of the classifier module in the test set and external validation set are shown in Fig. 5 [AUC 0.993 (95%CI: 0.978–0.999) in the test set for healthy patients vs. patients with acute pancreatitis, *P* < 0.001]; [AUC 0.850 (95%CI: 0.790–0.898) in the external validation set for healthy patients vs. patients with acute pancreatitis), *P* < 0.001]. The AUC of the classifier module in the test set and external validation set are detailed in Table 4.

**Table 3:** The training result of EasyDL

Label Name	Number of test sets	Precision (%)	Recalling rate (%)	f1-score (%)
Normal pancreas	142	100.0	99.0	99.0
Acute pancreatitis	210	99.1	100.0	100.0

**Table 4:** AUC of classifier module in test set and external validation set

Projects	Test set	External validation set
Sample size	352	186
Normal pancreas	210(59.66%)	94(50.54%)
Acute pancreatitis	140(40.34%)	92(49.46%)
AUC	0.993	0.850
Standard Errors	0.00496	0.0261
95% Confidence interval	0.978 to 0.999	0.790 to 0.898
z statistic	99.348	13.395
Significance level P (Area = 0.5)	< 0.0001	< 0.0001
Youden indx J	0.9859	0.6998
Sensitivity	100.00	80.85
Specificity	98.59	89.13

### Lesion segmentation module for identifying acute pancreatitis

#### Pancreas segmentation module

**Fig. 6:**
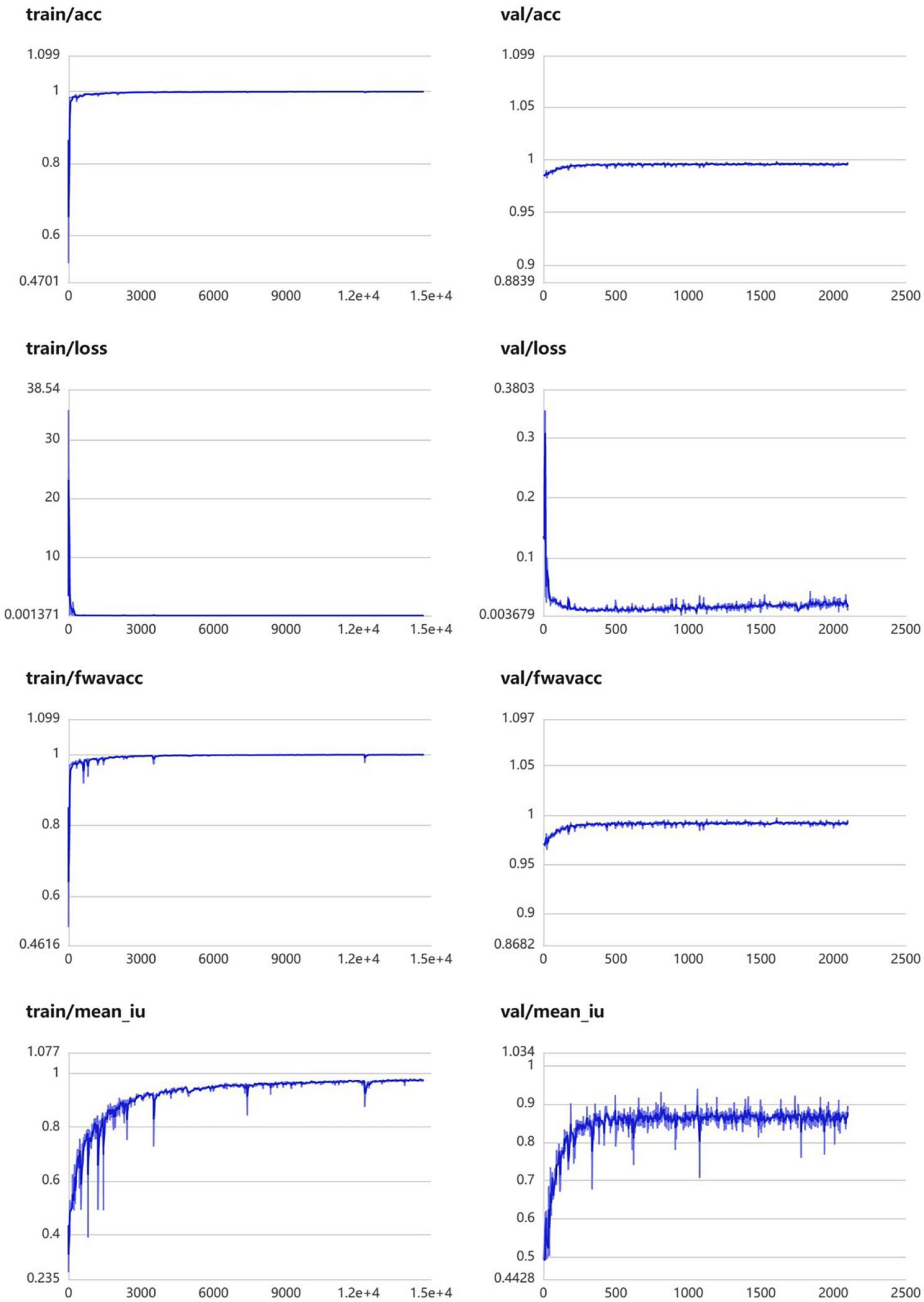
Accuracy, loss rate, fwavacc, and MIOU plots of the training and validation sets of the pancreas segmentation module

**Fig. 7:**
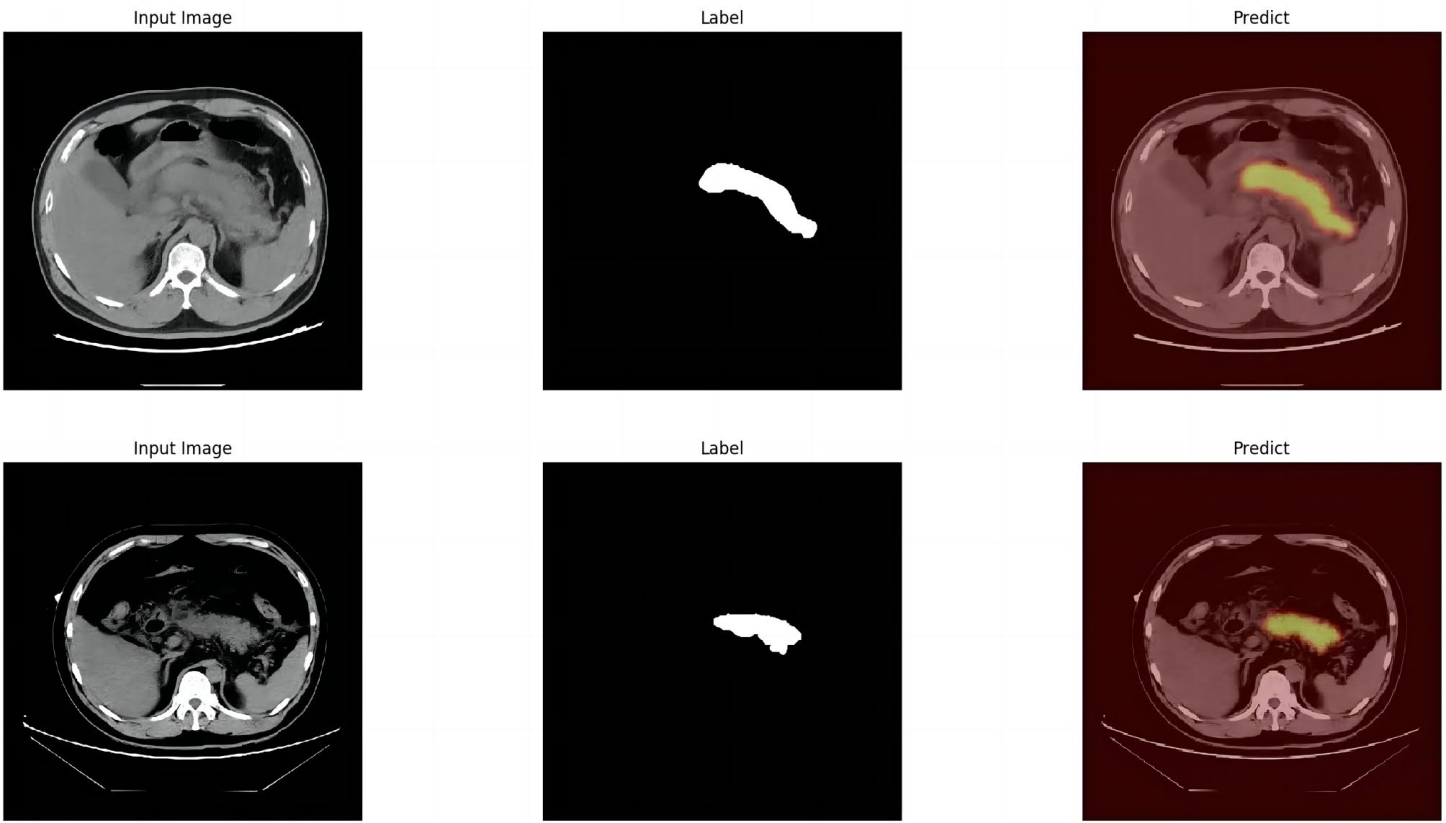
Effect plots of the pancreas segmentation module

The module was constructed to distinguish swollen and normal pancreas, using 675 segmentation images of swollen pancreas and 500 segmentation images of normal pancreas. The training parameters of this module were EPOCH-NUM = 300, BATCH-SIAZE = 16, train-num = 10. The module performed very well in pancreatic segmentation, and in the validation set, the median and quartiles of accuracy, loss rate, fwavacc, and mean cross-ratio were [99.54 (99.48, 99.59), 1.74 (1.36, 2.19), 99.14 (99.02, 99.23), 86.02 (84.52, 87.20)]. The accuracy, loss rate, fwavacc, and mean crossover sum of the training and validation sets of the module are shown in Fig. 6. The segmentation effects are shown in Fig. 7. The results of each parameter of the segmentation module in the validation set are shown in Table 5.

**Table 5:** The results of each parameter of the segmentation module in the validation set

Projects	Accuracy(%)	Loss(%)	Fwavacc(%)	MIOU(%)
pancreas	99.54(99.48, 99.59)	1.74(1.36, 2.19)	99.14(99.02, 99.23)	86.02(84.52, 87.20)
peripancreatic inflammatory exudate	99.06(98.89, 99.20)	3.60(2.85, 4.98)	98.29(97.97, 98.57)	61.81(56.25, 64.83)
peripancreatic effusion	98.86(98.40, 99.05)	4.07(3.12, 5.50)	97.79(96.91, 98.16)	57.73(49.90, 68.23)
peripancreatic abscess necrosis	97.94(97.64, 98.26)	4.80(4.29, 6.15)	96.36(95.51, 96.98)	66.36(55.08, 72.12)

#### Peripancreatic inflammatory exudate segmentation module

**Fig. 8:**
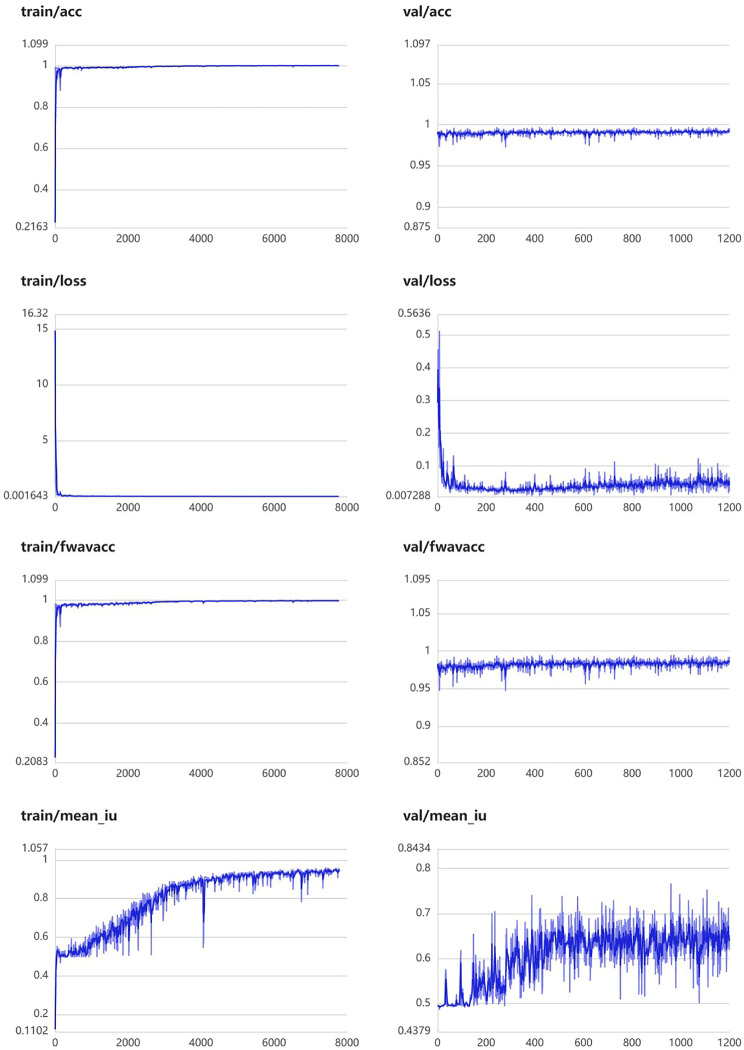
Accuracy, loss rate, fwavacc, and MIOU plots of the training and validation sets of the Peripancreatic inflammatory exudate segmentation module

**Fig. 9:**
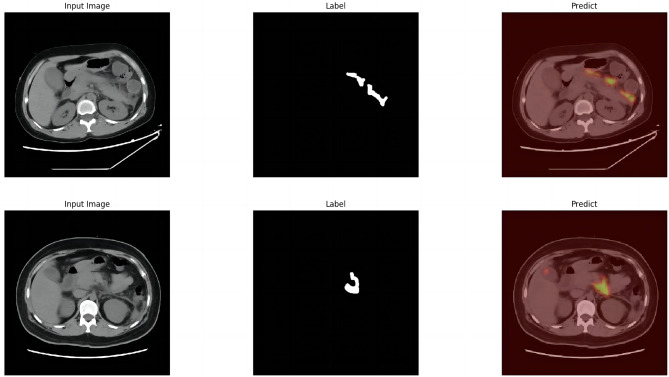
Effect plots of the Peripancreatic inflammatory exudate segmentation module

This module was constructed using 457 segmentation images of peripancreatic inflammatory exudate. The training parameters of this module were EPOCH-NUM = 300, BATCH-SIAZE = 16, train-num = 10. The module performed well in segmentation of peripancreatic inflammatory exudate, and in the validation set, the median and quartiles of accuracy, loss rate, fwavacc, and mean cross-comparison ratio were [99.06 (98.89, 99.20), 3.64 (2.85, 4.98), 98.29 (97.97, 98.57), and 61.81 (56.25, 64.83)]. The accuracy, loss rate, fwavacc, and mean crossover sum of the training and validation sets of the module are shown in Fig. 8. The splitting effect is shown in Fig. 9.

#### Peripancreatic effusion segmentation module

**Fig. 10:**
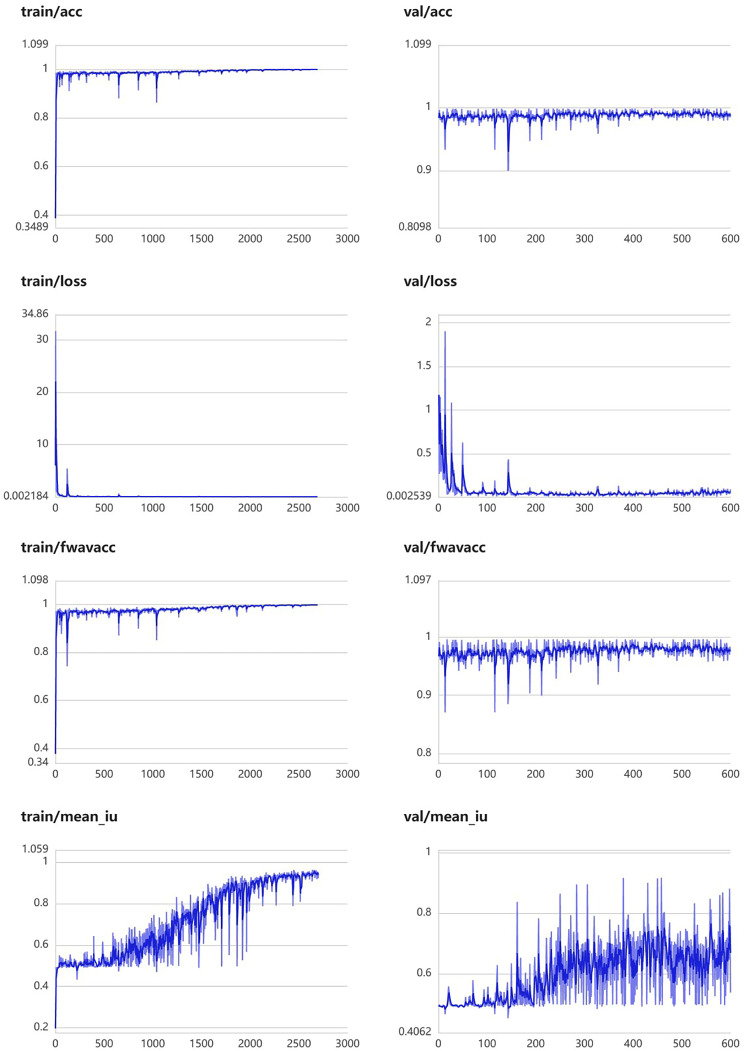
Accuracy, loss rate, fwavacc, and MIOU plots of the training and validation sets of the peripancreatic effusion segmentation module

**Fig. 11:**
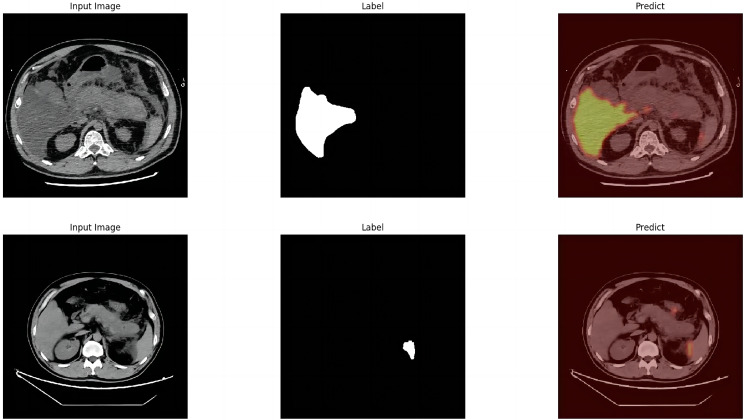
Effect plots of the peripancreatic effusion segmentation module

The peripancreatic effusion module was constructed using 153 segmentation images of peripancreatic effusion. The training parameters of this module were EPOCH-NUM = 400, BATCH-SIAZE = 16, train-num = 10. The module performed well in segmentation of peripancreatic inflammatory exudate, and in the validation set, the median and quartiles of accuracy, loss rate, fwavacc, and mean cross-comparison ratio were [98.86 (98.40, 99.05), 4.07 (3.12, 5.50), 97.79 (96.91, 98.16), 57.73 (49.90, 68.23)]. The accuracy, loss rate, fwavacc, and mean crossover sum of the training and validation sets of the module are shown in Fig. 10. The splitting effect is shown in Fig. 11.

#### Peripancreatic abscess necrosis segmentation module

**Fig. 12:**
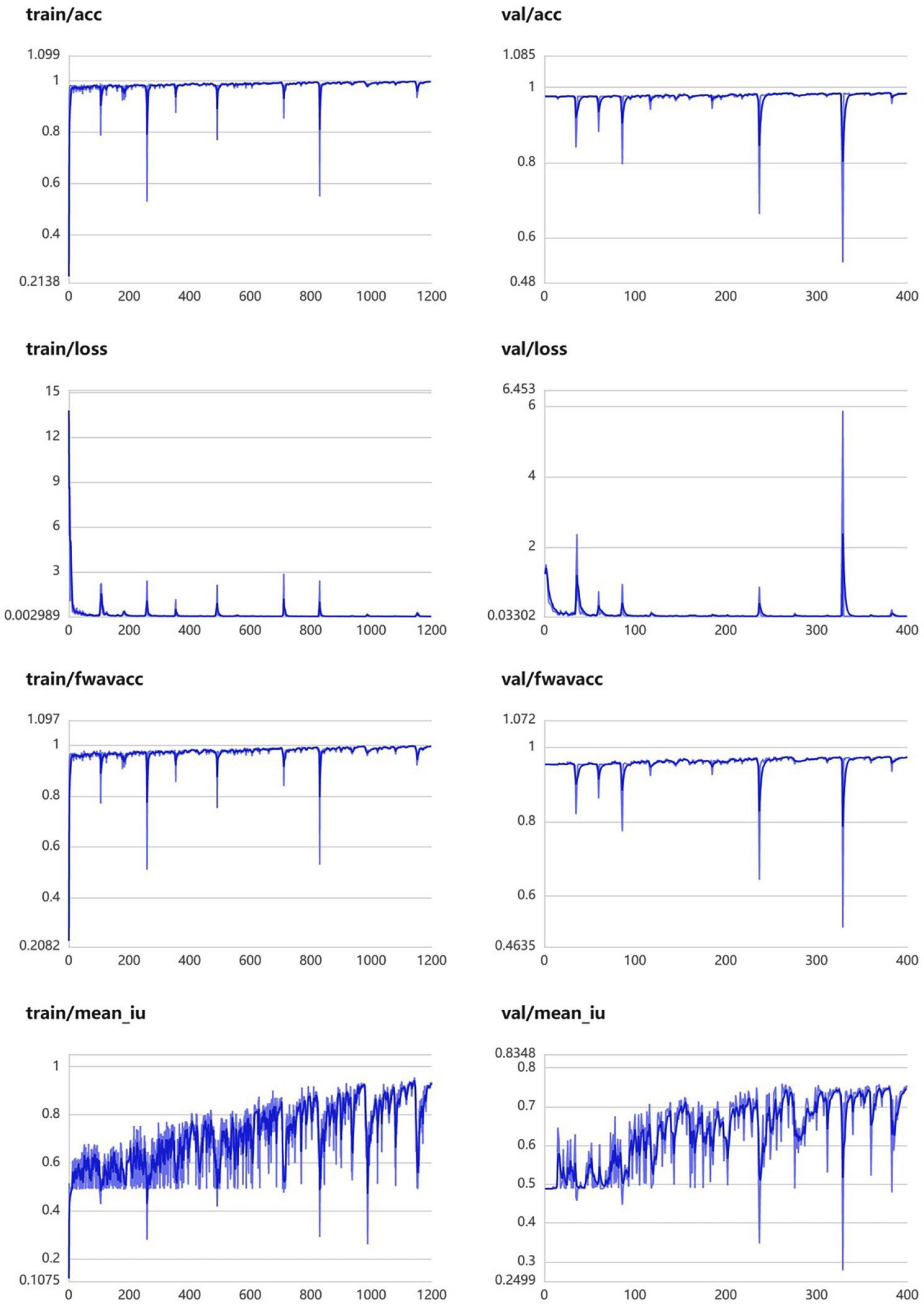
Accuracy, loss rate, fwavacc, and MIOU plots of the training and validation sets of the peripancreatic abscess necrosis segmentation module

**Fig. 13:**
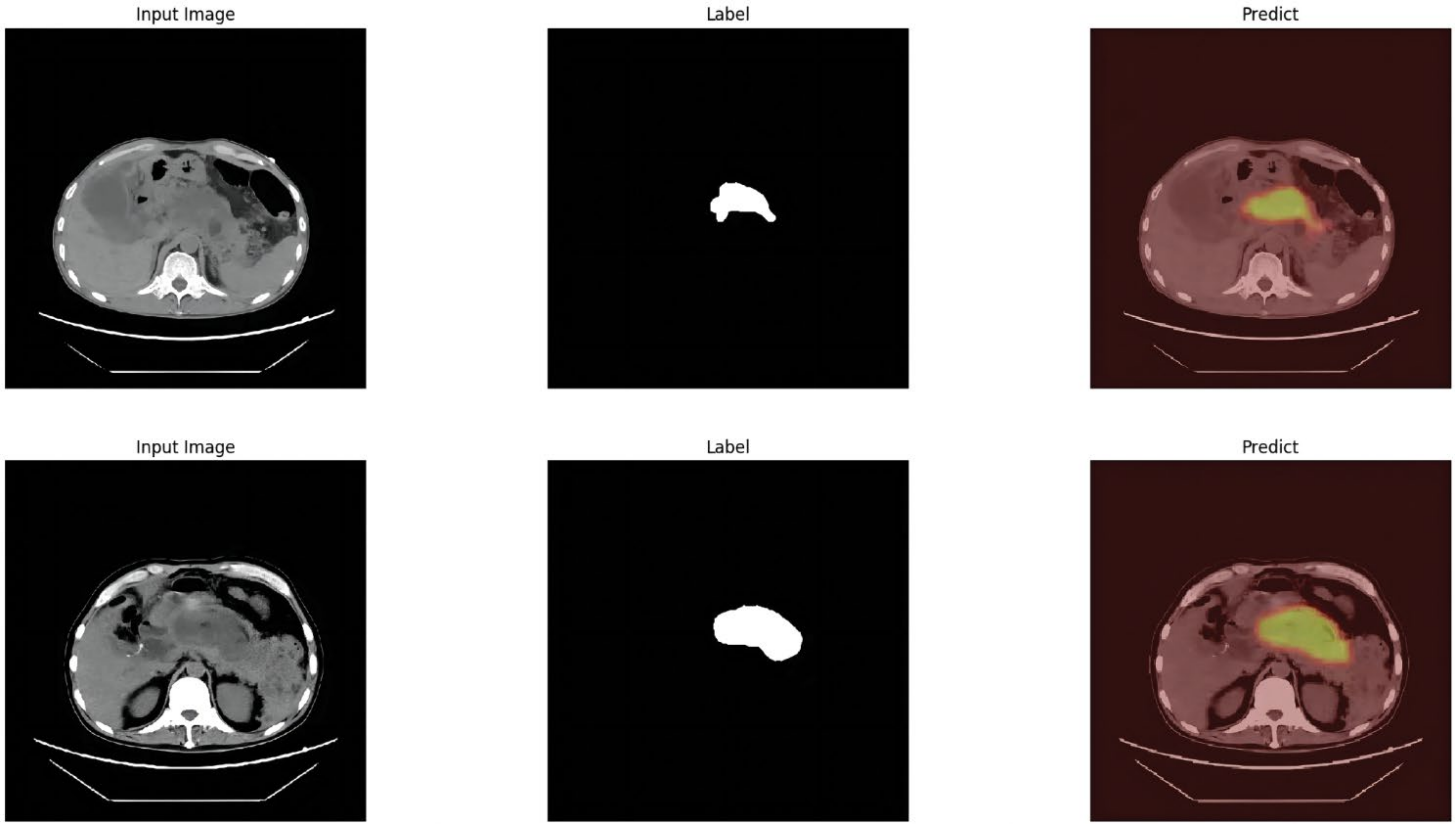
Effect plots of the peripancreatic abscess necrosis segmentation module

The peripancreatic abscess necrosis module was constructed using 42 segmentation maps of peripancreatic abscess necrosis. The training parameters of this module were EPOCH-NUM = 400, BATCH-SIAZE = 16, train-num = 10. The module performed well in segmentation of peripancreatic abscess necrosis, and in the validation set, the median and quartiles of accuracy, loss rate, fwavacc, and mean cross-comparison ratio were [97.94 (97.64, 98.26), 4.80 (4.29, 6.15), 96.36 (95.51, 96.98), 66.36 (55.08, 72.12)]. The accuracy, loss rate, fwavacc, and mean crossover sum of the training and validation sets of the module are shown in Fig. 12. The splitting effect is shown in Fig. 13.

## Discussion

In this study, we constructed a deep learning-powered diagnostic model for acute pancreatitis, which was able to effectively recognize acute pancreatitis and assess its severity by segmenting out the relevant lesions. The acute pancreatitis classifier module of this model showed high accuracy for the diagnosis of acute pancreatitis. In the test set [AUC of 0.993 (95% CI: 0.978–0.999), sensitivity of 100.00% and specificity of 98.59% for healthy patients vs. patients with acute pancreatitis]. In the external validation set [AUC of 0.850 (95% CI: 0.790–0.898), sensitivity of 80.85% and specificity of 89.13% for healthy patients vs. patients with acute pancreatitis]. In the pancreatitis segmentation module of this model, its segmenting ability of acute pancreatitis related lesions was also good, MIOU on the validation set was as high as 86.02%. This indicates that our model can diagnose acute pancreatitis quickly and accurately. As a result, it can have a positive impact on clinical practice. For example, the model can be deployed in more primary hospitals, which can assist emergency physicians to diagnose acute pancreatitis quickly and accurately, reducing the misdiagnosis rate while increasing the success rate of patient treatment.

The acute pancreatitis classifier module of this model achieved satisfactory results in terms of AUC-ROC, sensitivity, and specificity in both the test set and the external validation set. The credibility of this module is increased by the heatmap. Traditional DL models are deficient in interpretability and many studies [42–44] treated DL models as black boxes. In our study we have applied heatmap based on Shapley value [45] to improve the interpretability. In the pancreatitis classifier module of, we know exactly which regions the model is transforming with high weights to obtain the final discriminative results by the heatmap. From the correct classification Fig. 4(1a-1d) and Fig. 4(1e-1h) we can see that the module classifies the peripancreatic area as a high weight region. In addition, Fig. 4(1i-1l) shows the incorrectly classified images, and the module classified these two normal pancreatic images as pancreatitis images based on the peripancreatic region. It is not difficult to see from the figure that the module incorrectly considers the residual stomach as a high-weighted region. It is known that this is difficult even for a well-trained imaging physician.

EasyDL is a DL platform developed by Baidu that facilitates the entire process of model creation, data uploading, training the model, and model release. The underlying layer of EasyDL integrates AutoDL and AutoML technologies to automatically identify the optimal network. The platform eliminates the need for non-professionals to engage in tedious network selection and hyper-parameter tuning when constructing DL networks. Furthermore, EasyDL provides a heatmap, constructed using the Pixel-wise Shapley Value technique, which enables the user to identify the focus area. This is the primary reason this platform was selected for the construction of the pancreatitis classifier module. However, as this platform is a commercial platform, its most significant drawback is that it is not possible to ascertain which network it employs for training purposes or the training process itself.

In terms of segmentation method for acute pancreatitis lesions, we established our model by distinguishing four pathological types such as swell pancreases, peripancreatic inflammatory exudate, peripancreatic effusion, and peripancreatic abscess necrosis. In general, the model runs well (Table 5), but the performance on peripancreatic inflammatory exudate, peripancreatic effusion, and peripancreatic abscess necrosis are not satisfactory. The reasons for this were analyzed in conjunction with the results of the module segmentation. Peripancreatic inflammatory exudate and peripancreatic effusion are randomly distributed around the pancreas. From the segmentation results of the two modules in the validation set, the segmentation module identifies not only our pre-segmented lesions, but also some small and scattered lesions, which is equivalent to increasing the denominator of the MIOU. On the other hand, although the manual segmentation of the lesion is done by an imaging physician, selection bias is inevitable. However, by looking at the segmentation results, we found that the constructed module can in fact correct the selection bias, which explains the low MIOU and good segmentation results. Therefore, for these two modules, we cannot evaluate the performance of the module simply based on the magnitude of the MIOU and should instead combine the segmentation effects to make a comprehensive analysis. This also confirms that the DL model, as mentioned in study by Meglič J et al. [46], is actually learning and not simply mimicking the training dataset. This is a significant breakthrough in the field of medical image segmentation. As for the segmentation module of pancreatic abscess necrosis, the lack of sample size is the main reason for the low MIOU of the module, but the present results achieved by 42 segmented images have already shown that the module itself is highly successful.

To our best knowledge, our model is the first one that can distinguish acute pancreatitis in CT images. In addition, our model provides a segmentation function that can distinguish acute pancreatitis lesions, which is also unprecedented. This intelligent diagnostic model can assist clinicians to quickly recognize and assess the severity of acute pancreatitis through the segmentation of related lesions in a clinical setting. In terms of processing image data, this research ensured the quality of the dataset through manual segmentation by well-trained imaging physicians.

Currently, DL techniques have been applied to medical image segmentation and have demonstrated expert performance. In Li’s study [47], a meta-learning approach based on frequency domain feature mixing was proposed, which achieved a new level of generalization in MRI segmentation of nasopharyngeal carcinoma, with MIOU of 75.74%. The Swin MoCo network, a momentum contrast learning network with a Swin Transformer backbone, proposed by Xu et al. [48], has been shown to improve parotid segmentation to 85.18% MIOU. In Wang’s study [49], the deep learning model based on the U-net network demonstrated efficacy in fully automated image segmentation of adenoid and airway of nasopharynx in children, with MIOU values of 86.28% and 86.32%, respectively. Similar findings [50, 51] have been reported in other medical image segmentation models, more details can be found in Table 6. From the results presented in the table, it can be concluded that optimizing the model architecture is an urgent problem to be solved if the objective is to further improve the segmentation performance of the model.

**Table 6:** Comparative results with related literature

Author, Year	Region of interest	Model	Data sources	Sample	MIOU(%)
This work	pancreas	U-net	manual segmentation	1175	86.02
Yin Li, 2024	nasopharyngeal carcinoma	MF-Net	manual segmentation	321	75.74
Zian Xu, 2024	parotid gland	Swin MoCo with transfer	manual segmentation	148	85.18
Aleksandra Dzieniszewska, 2024	Skin	ResNet18	ISIC 2018 dataset	-	88.0
Wang L, 2023	adenoid of nasopharynx	U-net	manual segmentation	52	86.28
	airway of nasopharynx				86.32
Shaojun Zhu, 2022	Pterygium	Phase-fusion PSPNet	manual segmentation	517	86.31
		Double phase-fusion PSPNet			86.57

Although the segmentation function of U-Net is powerful, the acceptance domain of convolution operation in CNN is limited by the size of convolution kernel, resulting in a lack of long-distance dependence [52]. Therefore, CNN-based methods often have obvious limitations when it comes to displaying remote relationships in modeling. This is also the reason U-Net cannot make further breakthroughs. Transformer [53] is a popular approach in natural language processing that has been shown to be effective in learning global contextual features in computer vision and has demonstrated superior portability to downstream tasks under large-scale pre-training. It has been successful in the field of machine translation and natural language processing [54]. Therefore, it is proposed that TransUNet uses a CNN encoder to obtain local features, and then merges the Transformer into a hybrid encoder in the U-Net down-sampling path to obtain global contextual features [55]. The use of U-net alone to segment the pancreas is problematic when the basic textural features of the pancreas are not obvious compared to the surrounding peripheral organs. By combining CNN, which is good at capturing local features, and Transformer, which is good at capturing surrounding features, we can obtain more accurate segmentation than any traditional methods. Although our method achieves some good results in the segmentation of acute pancreatitis lesions, the module still has much room for improvement, potentially by capturing the surrounding features through Transformer.

Among the models for DL, the application of appropriate preprocessing approaches to the data or model can frequently enhance the learning results. Such examples include noise reduction, data balancing, data enhancement, and model architecture optimization, among others. In medical imaging, noise may have multiple sources and may affect the ability of the model to learn meaningful features. The importance of noise reduction to improve segmentation accuracy was highlighted in a study [56]. We can explore similar techniques such as denoising self-encoders or wavelet-based methods to mitigate noise in CT images. Category imbalance is a prevalent issue in DL models, particularly in classification models. When the number of samples of different categories in a dataset varies greatly, the model may exhibit a tendency to predict most of the categories while ignoring a few, which may subsequently affect the overall performance of the model. Although our dataset does not exhibit significant imbalances, the techniques discussed in Singh’s Study [57] can be borrowed, albeit with different application scenarios. For instance, oversampling a limited number of classes or the generation of synthetic data may be employed to achieve a more balanced distribution of acute pancreatitis and healthy cases. The study by Vaisali Chandrasekar [58] emphasizes the significance of data enhancement in improving the generalization capacity of models. It is possible to apply a number of enhancement techniques to CT images, including rotation, flipping and cropping, in order to artificially expand the dataset and expose the model to a greater variety of situations. Finally, optimization of the model architecture is often important as well. A study [36] demonstrated the effectiveness of an improved U-Net architecture for a segmentation task. Although our study employed the U-Net architecture, we may wish to consider integrating selected elements, such as dense connections or pyramid pooling modules, with the aim of enhancing our lesion segmentation model.

Although our model performs well on both the training and validation sets, it is prudent to exercise caution when employing AI to diagnose and treat diseases in clinical practice. One study [59] has demonstrated the potential risks associated with the use of Computer-aided Detection or Diagnostic (CAD) in clinical settings. However, we still encourage clinicians to consider it as a tool to assist in diagnosis.

This study has some limitations. Firstly, it is a single-center study, which increases the risk of bias. Secondly, the data set included in this study met the model requirements, but not every patient with pancreatitis had local complications such as effusion or necrosis, so there was still a lack of sufficient data to construct a better segmentation module for effusion and necrosis in acute pancreatitis.

## Conclusion

This study presents an innovative approach to the construction of an intelligent diagnostic model for acute pancreatitis, employing a DL algorithm. The model is designed to assist clinicians in rapidly and accurately identifying the presence of pancreatitis and segmenting lesions associated with acute pancreatitis. The model assists clinicians in assessing the severity of the disease in a more intuitive manner and in developing appropriate treatment plans for patients. Furthermore, in future work, we will continue to optimize the network and incorporate patients’ laboratory data into the model based on the existing model to construct a more comprehensive diagnostic model.

## Data Availability

The source code and relevant data for this study can be accessed at https://github.com/dcpengjin/PancreaseCT. The code is protected under the MIT license. EasyDL is a commercial deep learning platform developed by Baidu. The underlying layer of EasyDL combines AutoDL and AutoML technologies to automatically obtain the optimal network. However, it is important to note that the platform has some limitations, including the inability to provide algorithms in certain cases.

## Abbreviations

AI: Artificial intelligence
ALB: Albumin
AMY: Amylase
APACHE II: Acute Physiological and Chronic Health Score II
AST: Aspartate transaminase
AUC: Area Under the Curve
BISAP: Acute Pancreatitis Severity Bed Side Index
Ca: Calcium
CNN: Convolutional Neural Networks
Cr: Creatinine
CRP: C-reactive protein
CT: Computed tomography
DL: Deep learning
fwavacc: frequency-weighted accuracy
Glu: Glucose
HCT: Hematocrit
LDH: Lactate dehydrogenase
LPS: Lipase
MCTSI: Modified Computed Tomography Severity Index Score
MIOU: Mean Intersection over Union
ML: Machine learning
MR: Magnetic Resonance
PCT: Procalcitonin
ROC: Receiver Operating Characteristic
TB: Total bilirubin
TC: Total cholesterol
